# Reassessment of alkaline phosphatase as serum tumor marker with high specificity in osteosarcoma

**DOI:** 10.1002/cam4.1022

**Published:** 2017-05-11

**Authors:** Seung Hyun Kim, Kyoo‐Ho Shin, Seong‐Hwan Moon, Jinyoung Jang, Hyo Song Kim, Jin‐Suck Suh, Woo‐Ick Yang

**Affiliations:** ^1^ Department of Orthopedic SurgeryYonsei University College of MedicineSeoulKorea; ^2^ Division of Medical OncologyDepartment of Internal MedicineYonsei Cancer CenterYonsei University College of MedicineSeoulKorea; ^3^ Department of Radiology and Research Institute of Radiological ScienceYonsei University College of MedicineSeoulKorea; ^4^ Department of PathologyYonsei University College of MedicineSeoulKorea

**Keywords:** Alkaline Phosphatase, High specificity, Osteosarcoma, Serum Tumor Marker

## Abstract

The goal of this study was to reassess serum alkaline phosphatase (ALP) as tumor marker in osteosarcoma. We retrospectively examined serum ALP levels at diagnosis, every therapeutic step (neoadjuvant chemotherapy, surgery, and adjuvant chemotherapy), metastasis, and follow‐up and analyzed the role of ALP as tumor marker in 210 osteosarcomas. The diagnostic performances of ALP were validated with pathology‐proven 899 other primary bone lesions. Elevated ALP at diagnosis was associated with inferior overall survival (OS) *(*Log Rank *P <* 0.001) and disease‐free survival *(*Log Rank *P = *0.005) and independently significant for OS in multivariate analysis (hazard ratio [HR]=2.12, *P *=* *0.032*)*. During therapy, the ALP level significantly changed according to therapeutic steps (*P < *0.001 for patients ≥15 years old*, P < *0.001 for patients <15 years old) and survival (*P = *0.015 for ≥15 years*, P = *0.002 for <15 years), and the response of ALP to therapy and survival were associated (*P = *0.042 for ≥15 years*, P = *0.036 for <15 years). Initial ALP level was linearly correlated with tumor burden (total tumor volume; *P = *0.016 for ≥15 years, bone tumor volume; *P = 0.012* for ≥15 years). The sensitivity and specificity of ALP on diagnosis were 53.2% (95% Confidence Interval [CI]: 0.475–0.586) and 90.1% (95% CI: 0.888–0.913). The sensitivity of ALP on metastasis was 53.2% (95% CI: 0.431–0.624), and the specificity was 78.2% (95% CI: 0.720–0.839) at15 months postoperative and 90.0% (95% CI: 0.824–0.952) at 3 years postoperative. Serum ALP was found to be a valuable tumor marker with high specificity in osteosarcoma.

## Introduction

Serum tumor makers have been used in clinical management for diverse cancers. These markers can be used in screening early malignancy, diagnosis, determining prognosis, monitoring response to therapy, and postoperative surveillance 1, 2. For sarcomas, alkaline phosphatase (ALP) and lactate dehydrogenase (LDH) in osteosarcoma 3, 4, 5, LDH in Ewing`s sarcoma 6, 7, and myoglobin in rhabdomyosarcoma 8 have been identified and reported as prognostic serum markers. However, the role of these serum markers as tumor markers for sarcoma has not been established.

ALP is a ubiquitous enzyme present in all tissues but is mainly concentrated in the liver, kidney, placenta, and bone 9. In the musculoskeletal system, ALP is abundant in osteoblasts and is considered to play a role in the mineralization of newly formed bone. Serum ALP is considered a bone‐forming marker 10, 11. and has been used to monitor primary bone lesions. In fact, elevated levels of ALP in severe primary bone lesions have been reported 3, 4, 12, 13, and the possible role of ALP as tumor marker in osteosarcoma has been suggested 5, 14. However, clinical values of elevated levels of ALP in those diseases have not been validated.

To be an ideal tumor marker, a serum marker should meet several clinical requirements:^15^ The marker should be sufficiently sensitive and specific to the tumor, directly reflect tumor burden, correlate with results of therapy, and be useful for postoperative surveillance. For integration of a tumor marker into clinical practice, the characteristics of the marker must be validated to determine whether they meet these clinical requirements. We examined serum levels of ALP at every therapeutic step and during follow‐up from diagnosis to the last postoperative surveillance, analyzed them with respect to other clinical factors, and reassessed ALP as a tumor marker for osteosarcoma usingcriteria for the clinical requirements listed above.

## Materials and Methods

### Patients

We retrospectively reviewed the medical records of 210 patients who had been treated for osteosarcoma between October 1988 and November 2013 in Severance Hospital (Seoul, Korea). We examined the levels of serum ALP at diagnosis, every therapeutic step (neoadjuvant chemotherapy, surgery, and adjuvant chemotherapy), metastasis, and every follow‐up and analyzed them according to oncologic outcomes and other clinical factors. To validate the diagnostic performance of ALP, we also reviewed the ALP level at presentation for 899 patients with other primary bone lesions, including 208 with malignant bone tumors, 565 with benign bone tumors, and 126 with osteomyelitis, all of which were pathologically confirmed between January 1987 and January 2014 at Severance Hospital. This study protocol was approved by Severance Hospital institutional review board. The clinical characteristics of the enrolled 210 osteosarcoma patients are listed in Table 1. Among the total study population, 197 (93.5%) patients received surgery and 13 (6.2%) patients were inoperable, and 186 (88.6%) patients received chemotherapy and 24 (11.4%) patients did not. Among these 186 patients, 169 patients received both neoadjuvant and adjuvant chemotherapy, four received only adjuvant chemotherapy, and 13 received palliative chemotherapy without surgery. Among the 169 patients, 81 (47.9%) patients were treated with a doublet regimen of intraarterial cisplatin and doxorubicin; 76 (45.0%) were treated with a triplet of intraarterial cisplatin, doxorubicin, and ifosfamide; and 12 (7.1%) were treated with other regimens. Histologic and oncologic outcomes of neoadjuvant chemotherapy were not significantly different between doublet and triplet regimens in our cohort 16.

**Table 1:** Clinical characteristics and prevalence of ALP elevation at diagnosis

Variables	*n* (%)	ALP at diagnosis			
		Elevation *n* (%)	Normal *n* (%)	NA *n*	*P*
Survival					
5‐year Survival	116 (55.2)	49 (43.0)	65 (57.0)	2	0.000
DOD	67 (31.9)	50 (78.1)	14 (21.9)	3	
CDF <5 years	12 (5.7)				
NED	1 (0.5)				
AWD	3 (1.4)				
DOC	11 (5.2)				
Metastasis rate					
Positive	70 (37.4)	43 (64.2)	24 (35.8)	3	0.001
Free	117 (62.6)	46 (39.3)	71 (60.7)	0	
Stage					
Localized	187 (89.0)	89 (47.6)	94 (50.3)	4	0.000
Metastatic	23 (11.0)	20 (90.9)	2 (9.1)	1	
Age	20.5 (3–71)^a^				
≥15	131 (62.4)	71 (55.5)	57 (44.5)	3	0.395
<15	79 (37.6)	38 (49.4)	39 (50.6)	2	
Sex					
Male	117 (55.7)	73 (64.0)	41 (35.0)	3	0.000
Female	93 (44.3)	36 (38.7)	55 (59.1)	2	
Size					
≥ 8 cm	132 (68.0)	79 (62.2)	48 (37.8)	5	0.000
< 8 cm	62 (32.0)	20 (32.3)	42 (67.7)	0	
NA	16				
Location					
Extremity	185 (88.1)	93 (51.7)	87 (48.3)	5	0.247
Axial and proximal femur	25 (11.9)	16 (64.0)	9 (36.0)	0	
Histologic grade					
Low	20 (9.5)	3 (15.0)	17 (85.0)	0	0.000
High	190 (90.5)	106 (57.3)	79 (42.7)	5	
Histology (High grade)					
Conventional					
Osteoblastic	88 (58.7)	55 (63.2)	32 (36.8)	1	0.008^b^
Chondroblastic	18 (12.0)	8 (44.4)	10 (55.6)	0	
Fibroblastic	7 (4.7)	0 (0.0)	7 (100.0)	0	
Mixed	24 (16.0)	12 (54.5)	10 (45.5)	2	
Nonconventional	13 (8.7)	5 (35.8)	8 (61.5)	0	
NA	42				
Huvos grade					
I and II	52 (32.7)	32 (64.0)	18 (36.0)	2	0.158
III and IV	107 (67.3)	54 (51.9)	50 (48.1)	3	
NA	27				
No chemotherapy	24				
Surgery (Operability)					
Operable	197 (93.8)	98 (51.0)	94 (49.0)	5	0.019
Inoperable	13 (6.2)	11 (84.6)	2 (15.4)	0	
Resection margin					
R0	182 (92.4)	89 (50.0)	89 (50.0)	4	0.303
R1 and R2	15 (7.6)	9 (64.3)	5 (35.7)	1	
Pathologic fracture					
Yes	12 (5.7)	4 (33.3)	8 (66.6)	0	0.156
No	198 (94.3)	105 (54.4)	88 (45.6)	5	
Intracapsular extension					
Yes	38 (22.8)	18 (48.6)	19 (51.4)	1	0.732
No	139 (77.2)	71 (51.8)	66 (48.2)	2	
NA	33				
ALP At diagnosis		109 (53.2)	96 (46.8)	5	
ALP at 1st metastasis^c^					
Elevation	33 (53.2)	22 (73.3)	8 (26.7)	3	0.086
Normal	29(46.8)	15 (51.7)	14 (48.3)	0	
NA	8				

ALP, Alkaline Phosphatase; NA, Not Available; DOD, Died of Disease; CDF, Continuously Disease Free; NED, No Evidence of Disease; AWD, Alive with Metastatic Disease; DOC, Died of Other Cause; R0, Negative Resection Margin; R1, Microscopically Positive Resection Margin; R2, Macroscopically Positive Resection Margin.

a Mean(minimum‐maximum).

b Calculated using Fisher`s exact test.

c 63 pulmonary metastasis and 7 local recurs.

### Methods of serum ALP assays

Serum ALP levels were quantified in international units (IU), and enzyme activity was measured by the p‐nitrophenyl phosphate method 17. Serum ALP levels in children are significantly elevated due to high skeletal growth velocity and rapid bone turnover rate 18. Therefore, serum ALP ranges of 60.0–300.0 IU/L for patients aged <15 years and 38.0–115.5 IU/L for patients aged ≥15 years were considered normal to account for age‐related changes in serum levels. Serum ALP levels were estimated as total enzyme rather than bone isoenzyme.

### Statistical analysis

*χ* ^2^ test and Fisher`s extract test (if needed) were used to analyze differences in the prevalence of ALP elevation at diagnosis according to clinical factors. The Kaplan–Meier estimate was used to compare overall survival (OS) and disease‐free survival (DFS) between groups with elevated and normal serum ALP level at presentation. Cox regression analyses were used to evaluate the prognostic ability of ALP elevation at presentation. Linear Mixed model (fixed model) was used to determine whether changes in ALP during treatments were associated with therapeutic steps and survival, and whether the response of serum ALP to treatments was associated with survival. Spearman correlation analysis was used to evaluate the relationship of ALP levels at presentation with tumor burden. Two‐way contingency table analysis was used to validate the diagnostic performance of ALP on diagnosis and metastasis. All successive data of serum ALP were separately analyzed in patients <15 years and ≥15 years because of their different reference ranges. All statistical analyses were performed using SPSS (version 20.0, SPSS, Inc., Chicago, IL). All *P*‐values were two‐tailed and a *P*‐value < 0.05 was considered significant.

## Results

### Prevalence of ALP elevation at presentation according to clinical factors

The overall prevalence of ALP elevation at diagnosis was 53.2%. There were no significant differences in the prevalence of ALP elevation with respect to age (*P = *0.395), tumor location (*P = *0.395), Huvos grade (*P = *0.158), resection margin (*P = *0.303), pathologic fracture at presentation (*P = *0.156), and intracapsular extension (*P = *0.732) in *χ* ^2^ test (Table 1). However, the prevalence of ALP elevation at diagnosis varied significantly based on sex (*P < *0.001), metastasis at presentation (*P < *0.001), histologic grade (*P < *0.001), size (*P < *0.001), and operability (*P = *0.019). When compared according to histology, the osteoblastic type (58.7%) showed a much higher prevalence of elevated ALP than chondroblastic (12.0%), fibroblastic (4.7%), mixed (16.0%), or nonconventional (8.7%) types (*P = *0.008). The prevalence of ALP elevation at diagnosis was also significantly associated with oncologic outcome; survival (*P < *0.001) and metastasis (*P = *0.001). The prevalence of ALP elevation at first metastasis was 53.2%, similar to that at diagnosis. There was no difference in the prevalence of ALP elevation at diagnosis between patients with elevated level of ALP at first metastasis and patients with normal ALP (*P = *0.086).

### Prognostication of ALP

**Figure 1:**
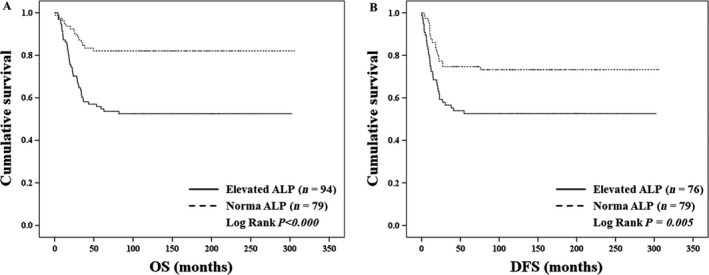
Survival analysis of patients with elevated alkaline phosphatase (ALP) at diagnosis and patients with normal ALP. (A) Kaplan–Meier analysis for overall survival (OS), including patients who were continuously disease free (CDF) for more than 5 years after surgery, with no evidence of disease (NED) for more than 5 years after last metastasectomy, and those who died of disease (DOD). The patients with elevated ALP level at presentation showed inferior OS *(Log Rank P < *0.001) compared with patients with normal ALP. (B) Kaplan–Meier analysis for disease‐free survival (DFS), which was analyzed for the patients with localized disease at presentation. The patients with elevated ALP level at presentation showed inferior DFS *(Log Rank P = *0.005) compared with patients with normal ALP.

The patients with elevated ALP level at presentation showed inferior OS *(Log Rank P < *0.001) and DFS *(Log Rank P = *0.005) compared with patients with normal ALP in Kaplan–Meier estimate (Fig. 1). Using Cox`s regression analysis, the prognostic value of ALP was compared with that of other prognostic factors (Table 2). The subclassifications of each prognostic factor were the same as those shown as Table 1. Metastasis at diagnosis (*P < *0.001), age (*P = *0.021), location (*P < *0.001), histologic grade (*P = *0.038), resection margin (*P < *0.001), and ALP (*P < *0.001) were significantly associated with OS in univariate analysis. ALP (*P = *0.032) showed independent association with OS after adjusting for metastasis at diagnosis (*P < *0.001), age (*P = *0.072), location (*P = *0.010), histologic grade (*P = *0.080), and resection margin (*P = *0.388). The statistical significance of ALP on OS even after adjusting for metastasis at diagnosis and histologic grade was quite remarkable. Regarding DFS, location (*P = *0.001), Huvos grade (*P = *0.007), resection margin (*P = *0.001), and ALP (*P = *0.006) were significantly associated in univariated analysis. However, ALP (*P = *0.126) was not independently associated with DFS and only Huvos grade (*P = *0.028) was significant after adjusting for these factors in multivariate analysis.

**Table 2:** Univariate and multivariate cox`s regression for prognostic factors

	Overall survival (*n = 183*)				Disease‐free survival (*n = 168*)			
	Univariate		Multivariate		Univariate		Multivariate	
	HR (95% CI)	*P*	HR (95% CI)	*P*	HR (95% CI)	*P*	HR (95% CI)	*P*
Metastasis at diagnosis	5.60 (3.27 to 9.61)	0.000	3.72 (1.89 to 7.30)	0.000				
Age	1.02 (1.00 to 1.04)	0.021	1.02 (1.00 to 1.04)	0.072	1.01 (0.99 to 1.03)	0.254	–	–
Size	1.63 (0.91 to 2.92)	0.101	–	–	1.66 (0.91 to 3.03)	0.101	–	–
Location	4.11 (2.40 to 7.03)	0.000	2.74 (1.27 to 5.90)	0.010	3.12 (1.58 to 6.18)	0.001	1.79 (0.73 to 4.44)	0.206
Histologic grade	8.05 (1.12 to 58.05)	0.038	6.15 (0.81 to 46.98)	0.080	3.60 (0.88 to 14.74)	0.075	–	–
Huvos grade	1.22 (0.67 to 2.22)	0.517	–	–	2.13 (1.23 to 3.71)	0.007	1.90 (1.07 to 3.37)	0.028
Resection margin	4.94 (2.60 to 9.40)	0.000	1.472 (0.61 to 3.55)	0.388	3.60 (1.70 to 7.62)	0.001	2.19 (0.82 to 5.81)	0.117
ALP	3.58 (1.98 to 6.48)	0.000	2.12 (1.07 to 4.21)	0.032	2.11 (1.23 to 3.62)	0.006	1.60 (0.88 to 2.93)	0.126

HR, Hazard Ratio; CI, Confidence Interval; ALP, Alkaline Phosphatase.

### Clinical characteristics of ALP with respect to clinical requirements of tumor marker

To validate the use of serum ALP to monitor the results of therapy, we analyzed changes in the ALP level throughout treatments. Among 169 patients who had received neoadjuvant, surgery, and adjuvant chemotherapy, 138 patients had good documentation of serum ALP values at diagnosis, after neoadjuvant therapy, after surgery, and after adjuvant chemotherapy. The serial measurements of ALP values from these 138 patients at the four therapeutic steps were analyzed by liner mixed modeling (Table 3). For patients with elevated ALP at diagnosis, of the ALP level varied significantly at each therapeutic step (*P < *0.001 in patients ≥15 years*, P < *0.001 in patients <15 years), suggesting that ALP respond to neoadjuvant chemotherapy, surgery, and adjuvant chemotherapy. The ALP level varied significantly at each therapeutic step in accordance with the outcomes of treatment (for 5‐year survival, *P = *0.015 in patients ≥15 years and *P = *0.002 in patients <15 years). Furthermore, the ALP level also varied significantly according to interactions between therapeutic steps and 5‐years survival (*P = *0.042 in patients ≥15 years*, P = *0.036 in patients <15 years), suggesting that response of ALP to treatment and patient survival were associated (Table 3). However, these changes were not significant in the patients with normal ALP at diagnosis.

**Table 3:** Linear mixed model analysis for serum levels of ALP during treatment^a^

	≥15 years old				<15 years old			
	Elevation (*n = 52)*		Normal (*n = 32*)		Elevation (*n = 24*)		Normal (*n = 30*)	
	E ± SE (95% CI)	*P*	E ± SE (95% CI)	*P*	E ± SE (95% CI)	*P*	E ± SE (95% CI)	*P*
Intercept	251.42 ± 30.85 (190.54–312.30)	0.000	82.89 ± 5.28 (72.41–93.37)	0.000	486.62 ± 65.42 (356.34–616.90)	0.000	191.31 ± 19.38 (152.83–229.80)	0.000
Treatment	−45.46 ± 11.30 (−67.80 to−23.11)	0.000	−1.67 ± 1.82 (−5.29 to 1.96)	0.364	−107.26 ± 24.65 (−156.61 to −57.92)	0.000	−15.51 ± 7.08 (−29.62 to −1.40)	0.032
Survival	104.15 ± 42.35 (20.58–187.72)	0.015	18.52 ± 13.46 (−8.18 to 45.23)	0.172	451.47 ± 143.80 (165.12–737.83)	0.002	58.83 ± 43.08 (−26.70 to 144.35)	0.175
Treatment * survival	−32.30 ± 15.72 (−63.39 to −1.21)	0.042	−8.96 ± 4.76 (−18.43 to 0.51)	0.063	−114.92 ± 53.47 (−221.98 to −7.85)	0.036	−24.34 ± 15.82 (−55.83 to 7.15)	0.128

E, Estimate; SE, Standard Error; CI, Confidence Interval; ALP, Alkaline Phosphatase.

a Analyzed by fixed model.

The ability to reflect tumor burden is another important clinical characteristic of a tumor marker. Tumors larger than 8 cm showed a higher prevalence of elevated ALP at presentation than tumors smaller than 8 cm (62.2% vs. 32.3%, *P = 0.000*) (Table 1). The initial tumor volumes on MRI were measured using an ellipsoid formula as previously described 19, 20. Total tumor volume and bone tumor volume were directly measured using ellipsoid formula and extended soft tissue tumor volume was indirectly calculated by subtracting bone tumor volume from total tumor volume. In Spearman correlation analysis, initial ALP level was linearly correlated with total tumor volume (*P = *0.016 in patients ≥15 years) and bone tumor volume (*P = *0.012 in patients ≥15 years), but not with extended soft tissue tumor volume (*P = *0.099 in patients ≥15 years), suggesting serum ALP reflect bone tumor volume (Table 4).

**Table 4:** Spearman correlation analysis between ALP and tumor size

	Total tumor volume		Bone tumor volume		Extended soft tissue tumor volume	
	Pearson correlation	*P*	Pearson correlation	*P*	Pearson correlation	*P*
ALP						
≥15 years (*n = 58*)	0.316	0.016	0.326	0.012	0.219	0.099
<15 years (*n = 42*)	−0.047	0.766	‐0.079	0.621	−0.038	0.812

ALP, Alkaline Phosphatase.

### Diagnostic performance of ALP at diagnosis and first metastasis

A tumor marker should be sufficiently sensitive and specific to exclude other malignant or benign diseases. To validate diagnostic performance of elevated ALP, the ALP levels at diagnosis of 899 other primary bone lesions were examined. Basic demographics of the 899 other primary bone lesions and their association with ALP elevation are listed in Table 5. The 899 lesions consisted of 208 malignant tumors, 565 benign tumors, and 126 cases of osteomyelitis, of which all were pathologically confirmed. (Table 6). Accuracy, sensitivity, and specificity of ALP among all primary bone lesions were 83.2% (95% CI: 0.811–0.853), 53.2% (95% CI: 0.475–0.586), and 90.1% (95% CI: 0.888–0.913), respectively. Although the sensitivity of ALP was not excellent, ALP was proven to be highly specific on diagnosis of osteosarcoma. Specificity of serum ALP for malignant tumors (88.9%) was similar to that for benign tumors (90.4%). The prevalence of ALP elevation at presentation was the highest for Ewing's sarcoma (26.1%) among malignant tumors and ossifying fibroma (21.4%) among benign tumors. Ewing`s sarcoma, undifferentiated pleomorphic sarcoma, giant cell tumor (GCT), fibrous dysplasia, Langerhans cell histiocytosis (LCH), and osteomyelitis may be often confused with osteosarcoma in imaging studies. In exclusive analysis with these tumor types, the accuracy, sensitivity, and specificity of ALP were 76.1% (95% CI: 0.726–0.792), 53.2% (95% CI: 0.483–0.575), and 89.2% (95% CI: 0.864–0.916), respectively. This high specificity of ALP may help to discriminate osteosarcoma and other tumor types.

**Table 5:** Basic demographics and prevalence of ALP Elevation at diagnosis of 899 primary bone lesions

	Sex				Age	
	ALP at diagnosis	M (%)	F (%)	*P*	Mean ± SD	Min‐Max
Malignancy						
CS	Elevation	5 (8.8)	2 (4.5)	0.465	45.4 ± 16.0	14–91
	Normal	52 (91.2)	42 (95.5)			
ES	Elevation	4 (33.3)	2 (18.2)	0.640	15.4 ± 8.6	1–38
	Normal	8 (66.7)	9 (81.8)			
UPS	Elevation	2 (20.0)	1 (11.1)	1.000	45.2 ± 19.1	16–71
	Normal	8 (80.0)	8 (88.9)			
CD	Elevation	2 (22.2)	1 (10.0)	0.582	48.4 ± 23.8	3–76
	Normal	7 (77.8)	9 (90.0)			
MM	Elevation	4 (15.4)	0 (0.0)	0.069	58.4 ± 10.8	34–84
	Normal	22 (84.6)	20 (100.0)			
Benign						
OC	Elevation	18 (15.9)	2 (3.4)	0.022	18.0 ± 11.5	1–63
	Normal	95 (84.1)	56 (96.6)			
EC	Elevation	4 (12.5)	0 (0.0)	0.009	35.6 ± 16.7	5–74
	Normal	28 (87.5)	69 (100.0)			
GCT	Elevation	4 (7.1)	3 (4.8)	0.705	32.6 ± 16.7	5–74
	Normal	52 (92.9)	60 (95.2)			
FD	Elevation	2 (6.7)	4 (16.0)	0.394	26.7 ± 14.4	2–63
	Normal	28 (93.3)	21 (84.0)			
SBC	Elevation	1 (6.3)	0 (0.0)	1.000	23.1 ± 15.5	5–66
	Normal	15 (93.8)	7 (100.0)			
ABC	Elevation	2 (40.0)	0 (0.0)	0.111	16.1 ± 10.6	5–48
	Normal	3 (60.0)	9 (100.0)			
OF	Elevation	3 (50.0)	0 (0.0)	0.055	10.9 ± 7.4	1–22
	Normal	3 (50.0)	8 (100.0)			
NOF	Elevation	1 (25.0)	0 (0.0)	1.000	13.9 ± 4.1	5–17
	Normal	3 (75.0)	3 (100.0)			
CB	Elevation	5 (26.3)	0 (0.0)	0.278	20.6 ± 9.5	5–47
	Normal	14 (73.7)	7 (100.0)			
OO	Elevation	0 (0.0)	0 (0.0)	ND	18.5 ± 10.6	3–46
	Normal	14 (100.0)	2 (100.0)			
LCH	Elevation	0 (0.0)	1 (11.1)	0.474	9.0 ± 9.3	1–32
	Normal	10 (100.0)	8 (88.9)			
OM	Elevation	13 (16.3)	3 (6.5)	0.165	37.3 ± 22.4	1–85
	Normal	67 (83.8)	43 (93.5)			

ALP, Alkaline Phosphatase; OS, Osteosarcoma; CS, Chondrosarcoma; EW, Ewing`s sarcoma; PS, Undifferentiated Pleomorphic Sarcoma (Malignant Fibrous Histiocytoma); CD, Chordoma; MM, Multiple Myeloma/Plasmacytoma; ST, Subtotal; OC, Osteochondroma; EC, Enchondroma; GCT, Giant Cell Tumor; FD, Fibrous Dysplasia; SBC, Simple Bone Cyst; ABC, Aneurysmal Bone Cyst; OF, Ossifying Fibroma; NOF, Nonossifying fibroma; CB, Chondroblastoma; OO, Osteoid Osteoma; LCH, Langerhans Cell Histiocytosis; OM, Osteomyelitis.

**Table 6:** Two‐way contingency table analysis for performance of ALP on diagnosis

	OS	Nonosteosarcoma																			
		Malignant lesions						Benign lesions													Total
		CS	EW	PS	CD	MM	ST	OC	EC	GCT	FD	SBC	ABC	OF	NOF	CB	OO	LCH	OM	ST	
Elevated ALP *n* (%)	109 (53.2)	7 (6.9)	6 (26.1)	3 (15.8)	3 (15.8)	4 (8.7)	23 (11.1)	20 (11.7)	4 (4.0)	7 (5.9)	6 (10.9)	1 (4.3)	2 (14.3)	3 (21.4)	1 (14.3)	5 (19.2)	0 (0.0)	1 (5.3)	16 (12.7)	66 (9.6)	89 (9.9)
Normal ALP *n* (%)	96 (46.8)	94 (93.1)	17 (73.9)	16 (84.2)	16 (84.2)	42 (91.3)	185 (88.9)	151 (88.3)	97 (96.0)	112 (94.1)	49 (89.1)	22 (95.7)	12 (85.7)	11 (78.6)	6 (85.7)	21 (80.8)	16 (100.0)	18 (94.7)	110 (87.3)	625 (90.4)	810 (90.1)
Total (*n*)	205	101	23	19	19	46	208	171	101	119	55	23	14	14	7	26	16	19	126	691	899
Accuracy % (95% CI)		71.2 (0.67–0.75)						81.9 (0.79–0.84)													83.2 (0.81–0.85)
Sensitivity % (95% CI)		53.2 (0.49–0.57)						53.2 (0.48–0.58)													53.2 (0.48–0.59)
Specificity % (95% CI)		88.9 (0.85–0.92)						90.4 (0.89–0.92)													90.1 (0.89–0.91)
PPV % (95% CI)		82.6 (0.76–0.88)						62.3 (0.56–0.68)													55.1 (0.49–0.61)
NPV % (95% CI)		65.8 (0.63–0.68)						86.7 (0.85–0.88)													89.4 (0.88–0.91)
PLR (95% CI)		4.81 (3.21–7.41)						5.57 (4.28–7.23)													5.37 (4.24–6.76)
NLR (95% CI)		0.53 (0.47–0.60)						0.52 (0.45–0.59)													0.52 (0.45–0.59)
DOR (95% CI)		9.13 (5.32–15.79)						10.75 (7.28–15.91)													10.33 (7.17–14.91)

ALP, Alkaline Phosphatase; OS, Osteosarcoma; CS, Chondrosarcoma; EW, Ewing`s sarcoma; PS, Undifferentiated Pleomorphic Sarcoma (Malignant Fibrous Histiocytoma); CD, Chordoma; MM, Multiple Myeloma/Plasmacytoma; ST, Subtotal; OC, Osteochondroma; EC, Enchondroma; GCT, Giant Cell Tumor; FD, Fibrous Dysplasia; SBC, Simple Bone Cyst; ABC, Aneurysmal Bone Cyst; OF, Ossifying Fibroma; NOF, Nonossifying fibroma; CB, Chondroblastoma; OO, Osteoid Osteoma; LCH, Langerhans Cell Histiocytosis; OM, Osteomyelitis; CI, Confidence Interval; PPV, Positive Predictive Value; NPV, Negative Predictive Value; PLR, Positive Likelihood Ratio; NLR, Negative Likelihood Ratio; DOR, Diagnostic Odds Ratio.

The performance of ALP elevation on first metastasis was also validated in both the early postoperative phase that is prone to metastasis and late stable postoperative phase (Table 7). Among 70 patients with metastasis, 62 had records for serum ALP on first metastasis, of which 57 were presented as pulmonary metastasis without local recur and five as local recurrence. Because the mean latency to first metastasis in this study cohort was 15.1 ± 13.2 months, we analyzed ALP level at approximately 15 months postoperative and also at approximately 3 years postoperative to monitor the stable phase. The mean follow‐up periods for ALP were 14.0 ± 4.0 months for 15 months postoperative and 35.1 ± 4.8 months for 3 years postoperative. The sensitivity of ALP upon metastasis was 53.2% (95% CI: 0.431–0.624), similar to that on diagnosis. In early phase, the specificity of serum ALP for metastasis declined to 78.2% (95% CI: 0.720–0.839). On the other hand, specificity of serum ALP for metastasis increased to 90.0% (95% CI: 0.824–0.952) in late stable phase. On the basis of these findings, ALP should be considered as a supportive method for monitoring metastasis in addition to regular imaging at short intervals during the early phase. However, ALP may be an efficient means of monitoring during the longer intervals between imaging in the late stable phase.

**Table 7:** Two‐way contingency table analysis for performance of ALP on metastasis

	15 months postoperative^a^			3 years postoperative^a^		
	Metastasis positive^b^	Metastasis free	Total	Metastasis positive^b^	Metastasis free	Total
ALP at Metastasis						
Elevation *N* (%)	33 (53.2)	22 (21.8)	55 (33.7)	33 (53.2)	7 (10.0)	40 (30.3)
Normal *N* (%)	29 (46.8)	79 (78.2)	108 (66.3)	29 (46.8)	63 (90.0)	92 (69.7)
Total	62	101	163	62	70	132
Accuracy % (95% CI)	68.7 (0.610–0.757)			72.7 (0.646–0.783)		
Sensitivity % (95% CI)	53.2 (0.431–0.624)			53.2 (0.446–0.591)		
Specificity % (95% CI)	78.2 (0.720–0.839)			90.0 (0.824–0.952)		
PPV % (95% CI)	60.0 (0.486–0.704)			82.5 (0.691–0.917)		
NPV % (95% CI)	73.1 (0.673–0.784)			68.5 (0.627–0.725)		
PLR (95% CI)	2.444 (1.539–3.869)			5.323 (2.529–12.436)		
NLR (95% CI)	0.598 (0.448–0.790)			0.520 (0.429–0.673)		
DOR (95% CI)	4.086 (1.947–8.634)			10.241 (3.760–28.992)		

ALP, Alkaline Phosphatase; CI, Confidence Interval; PPV, Positive Predictive Value; NPV, Negative Predictive Value; PLR, Positive Likelihood Ratio; NLR, Negative Likelihood Ratio; DOR, Diagnostic Odds Ratio; SD, Standard Deviation.

Latency to metastasis, months (Mean ± SD): 15.1 ± 13.2.

ALP follow‐up period, months (Mean ± SD): 14.0 ± 4.0.

a ALP follow‐up period, months (Mean ± SD): 35.1 ± 4.8.

b 57 pulmonary metastasis and 5 local recurs.

## Discussion

The prevalence of serum ALP elevation at diagnosis of osteosarcoma in our cohort was 53.2% (Table 1). The prevalence of ALP elevation at diagnosis varied significantly based on sex (*P < *0.001), metastasis at presentation (*P < *0.001), histologic grade (*P < *0.001), size (*P < *0.001), and operability (*P = *0.019). All these factors were independently associated in multivariate logistic regression analysis *(data not shown)*. Unlike other tumor‐related factors, sex was the only trait that affected ALP level in osteosarcoma patients. Male gender was significantly associated with higher prevalence of ALP elevation in our cohort, but did not lead to an association with survival (*P = *0.054) and metastasis (*P = *0.076). Several large‐scaled studies reported disaccording results for associations between sex and prevalence of ALP elevation at presentation. *Han* et al.*,* reported that there was no association between sex and prevalence of ALP elevation in their cohort of 177 osteosarcoma patients in southern China 21. Rizzoli group reported two contrary results between 741 osteosarcoma patients enrolled from March 1972 to December 1989^4^ and 560 osteosarcoma patients enrolled from March 1983 to June 1955^22^. No association was found between sex and prevalence of ALP elevation in the former cohort, although male gender was significantly associated in the latter. However, sex was not associated with oncologic outcomes in both cohorts.

The prevalence of elevated ALP at presentation has been reported to range from 31.5% to 66.3% 4, 23, 24, 25, 26, 27. The main difference among the results seemed to be caused by the distribution of metastatic stages in each study cohort. Indeed, the prevalence of ALP elevation at diagnosis in the metastatic stage was extremely high: 90.5% in this study (Table 1) and 91.5% in a previous report 4. Studies that excluded metastatic stage showed a lower prevalence (37.2%^25^ and 47.0%^26^) than studies that included metastatic stage (51.2%^23^, 58.5%^27^, and 66.3%^4^). Given these findings, the sensitivity of ALP on diagnosis in our cohort (53.2%) may be generalizable.

Basic demographic factors of 899 patients with primary bone lesion, as well as their association with the prevalence of elevated ALP, are listed in Table 5. Higher prevalence of ALP elevation at presentation in male was observed in most tumors; however, significant association was found only in osteochondroma and enchondroma. Mean onset ages of most benign tumors and Ewing`s sarcoma were under 20 years, whereas those of other malignant tumors were over 40 years. All 899 patients received medical examination for anesthesia and operability. In respect to underlying diseases in 91 patients with elevated ALP, pulmonary tuberculosis was most common in seven patients, hypertension in three, diabetes and hypothyroidism in two, epilepsy and COPD in one. A total of ten patients showed elevated levels of either serum aspartate aminotransferase or alanine aminotransferase. Only one of them showed accompanied elevation in total serum bilirubin level, suggesting hepatobiliary disease that can affect serum ALP level, and three were proven as viral hepatitis.

The specificity of ALP on diagnosis of osteosarcoma has not been previously reported. In comparisons with 899 other primary bone lesions, ALP showed excellent specificity of 90.1% in this study; 88.9% among malignant lesions and 90.4% among benign lesions (Table 5). The sensitivity and specificity of ALP on metastasis during postoperative surveillance is another important characteristic of a tumor marker. The sensitivity of ALP on first metastasis was 53.2% and the specificity were 78.2% in the early metastasis‐prone stage and 90.0% in late stable stage.

Compared with well‐established tumor markers in other cancers, ALP in osteosarcoma showed similar sensitivity on diagnosis with alpha‐fetoprotein in hepatocellular carcinoma 28, 29 and carcinoembryonic antigen (CEA) and cytokeratin 19 fragment (CYFRA‐21) in lung cancer 30, 31, 32, for which sensitivity ranged from 39.0% to 68.6%. On the other hand, the sensitivity of ALP on diagnosis was inferior to that prostate‐specific antigen in prostate cancer 33, CA 125 in epithelial ovarian cancer 34, 35, 36, and CA 19–9 in pancreatic cancer 37, 38, 39, for which sensitivity ranged from 71.9% to 89.3%, but superior to that of CA 19–9 and CEA in colorectal cancer 40, and CA 15–3 in breast cancer 41, 42, which show sensitivity ranging from 15.4% to 31.7%. The sensitivity and specificity of ALP on metastasis during postoperative surveillance were similar to those of CA 19–9 (sensitivity: 69.0% and specificity: 94.5%) and CEA (sensitivity: 47.1% and specificity: 95.7%) in colorectal cancer 40. Overall, the diagnostic performance of ALP on diagnosis and metastasis in osteosarcoma was found to compare favorably with well‐known tumor markers in other cancers.

In this study, elevated serum ALP level at presentation was poorly associated with both OS and DFS; however, results from multivariate analysis of our cohort showed that ALP was independently associated with OS except for DFS. This may cause doubts regarding prognostic performance of ALP for metastasis or recurrence. However, according to two recent reports of meta‐analyses for ALP and its prognosis for osteosarcoma, high serum ALP level was significantly associated with both OS and DFS poorly 43, 44 The discrepancy between this study and the mentioned meta‐analyses seems to be due to selection bias, which could have been caused by our study cohort having an unusually strong association with chemosensitivity and metastasis, rather than with prognostic performance of ALP for metastasis or recurrence itself. Therefore, we expect ALP to be useful for predicting metastasis or recurrence, as well as survival.

Unlike other cancers, there are some unique situations regarding the management of osteosarcoma, such that in the clinic, high specificity of a tumor marker is more useful than high sensitivity. First, osteosarcoma can be easily screened by plain X‐ray, so there is less need for a tumor marker with high sensitivity for tumor screening. Second, MRI and nuclear medicine imaging provide sufficiently sensitive information suggestive of osteosarcoma. Indeed, in the clinical situation, osteosarcoma is more likely to be confused with other primary bone lesions than to be missed. In this difficult situation, the high specificity of ALP may be very useful. Third, recurrence of osteosarcoma is mainly presented as pulmonary metastasis rather than local recur, so that chest CT is essential for postoperative surveillance. Chest CT is usually followed at short intervals in early postoperative surveillance because most metastasis occurs within 2 years postoperative. However, chest CT is followed at long intervals in the late postoperative surveillance because of the decreased metastasis incidence of metastasis and the risk of radiation hazard. The specificity of ALP on metastasis recovered by 90.0% at 3 years postoperative (Table 6), such that ALP elevation during the long intervals between chest CT in late postoperative surveillance is strongly suggestive of development of metastasis. ALP may therefore be useful to bridge the gap between chest CT scans.

ALP has been proven to be a prognostic factor in osteosarcoma. However, it is not clear whether the increased production of ALP in osteosarcoma is the result of osteoblastic tumor cells or reactive bone formation in response to osteolysis by the tumor. This study did not provide direct evidence for either possibility, but indirect evidences favoring production of ALP by tumor cells. First, the osteoblastic type showed prominent prevalence of ALP elevation at diagnosis compared with other histological types (Table 1). Second, ALP was also elevated on pulmonary metastasis without local recur (49.1%) with a similar prevalence to that at diagnosis (53.1%). Third, initial ALP level correlated with tumor burden and stage. Forth, in primary benign osteolytic tumors such as giant cell tumor (GCT), simple bone cyst (SBC), aneurysmal bone cyst (ABC), nonossifying fibroma (NOF), and Langerhans cell histiocytosis, in which osteogenesis by the tumors is seldom observed in pathology, the prevalence of ALP elevation at diagnosis was only 7.1%, which may be considered the result of reactive bone formation (Table 6).

Our study had some limitations. First, it was a retrospective study over a long time span. Second, in validating the diagnostic performance of ALP, primary bone lesions other than osteosarcoma were biased toward certain lesions. Malignant tumors weighed heavily in favor of chondrosarcoma according to incidence and benign lesions weighed in favor of osteochondroma, enchondroma, GCT, and osteomyelitis according to the stage. In fact, although SBC, ABC, and NOF were common, the number of pathologically confirmed cases was very small because most were in an inactive stage. Third, ALP levels were estimated as total enzyme rather than bone isoenzyme, which is theoretically specific to bone. However, the bone isoenzyme predominates in childhood. In adults the bone and liver isoenzymes are present in an equal amount with the intestinal isoenzyme accounting for <10%, and the bone isoenzyme may be also increased in adults with liver disease because it is cleared by the liver. For this reason, it is not clear whether bone isoenzyme is really superior to total enzyme for representing bone formation activity in the clinical setting 10, 45. New candidate tumor markers continue to be reported; however, only a small number of these have been successfully integrated into clinical practice because most lack evidence for clinical value. Clinical value of a marker should be validated on the basis of sensitivity, specificity, correlation with tumor burden, and responsiveness to therapy. Using these criteria, ALP was found to be a valuable tumor marker with high specificity in osteosarcoma, and is the first validated tumor marker in sarcomas.

## Conflict of Interests

The authors declare that they have no competing interests.
